# Transgenerational Inheritance of Modified DNA Methylation Patterns and Enhanced Tolerance Induced by Heavy Metal Stress in Rice (*Oryza sativa* L.)

**DOI:** 10.1371/journal.pone.0041143

**Published:** 2012-09-11

**Authors:** Xiufang Ou, Yunhong Zhang, Chunming Xu, Xiuyun Lin, Qi Zang, Tingting Zhuang, Lili Jiang, Diter von Wettstein, Bao Liu

**Affiliations:** 1 Key Laboratory of Molecular Epigenetics of MOE and Institute of Genetics and Cytology, Northeast Normal University, Changchun, China; 2 Jilin Academy of Agricultural Sciences, Changchun, China; 3 Department of Crop and Soil Sciences and School of Molecular Biology, Washington State University, Pullman, Washington, United States of America; National Taiwan University, Taiwan

## Abstract

**Background:**

DNA methylation is sensitive and responsive to stressful environmental conditions. Nonetheless, the extent to which condition-induced somatic methylation modifications can impose transgenerational effects remains to be fully understood. Even less is known about the biological relevance of the induced epigenetic changes for potentially altered well-being of the organismal progenies regarding adaptation to the specific condition their progenitors experienced.

**Methodology/Principal Findings:**

We analyzed DNA methylation pattern by gel-blotting at genomic loci representing transposable elements and protein-coding genes in leaf-tissue of heavy metal-treated rice (*Oryza sativa*) plants (S0), and its three successive organismal generations. We assessed expression of putative genes involved in establishing and/or maintaining DNA methylation patterns by reverse transcription (RT)-PCR. We measured growth of the stressed plants and their unstressed progenies *vs.* the control plants. We found (1) relative to control, DNA methylation patterns were modified in leaf-tissue of the immediately treated plants, and the modifications were exclusively confined to CHG hypomethylation; (2) the CHG-demethylated states were heritable via both maternal and paternal germline, albeit often accompanying further hypomethylation; (3) altered expression of genes encoding for DNA methyltransferases, DNA glycosylase and *SWI/SNF* chromatin remodeling factor (*DDM1*) were induced by the stress; (4) progenies of the stressed plants exhibited enhanced tolerance to the same stress their progenitor experienced, and this transgenerational inheritance of the effect of condition accompanying heritability of modified methylation patterns.

**Conclusions/Significance:**

Our findings suggest that stressful environmental condition can produce transgenerational epigenetic modifications. Progenies of stressed plants may develop enhanced adaptability to the condition, and this acquired trait is inheritable and accord with transmission of the epigenetic modifications. We suggest that environmental induction of heritable modifications in DNA methylation provides a plausible molecular underpinning for the still contentious paradigm of inheritance of acquired traits originally put forward by Jean-Baptiste Lamarck more than 200 years ago.

## Introduction

Being sessile, plants are constantly exposed to myriads of environmental stress conditions, and to which they are initially maladapted. Thus, a stress condition often has negative effect on organism growth, development, and reproduction. Depending on their nature, stresses can be divided into internal and external, with external stresses being further subdivided into two classes: biotic and abiotic stresses [1]. Excessive or inadequate light, water, salt, temperature and heavy metal all constitute abiotic stresses. It has been documented that plants have evolved sophisticated modulating mechanisms to respond and adapt to the various stress conditions. These modulating mechanisms operate at various levels, including short-term physiological, metabolic and gene expression changes, and long-term genetic and epigenetic modifications of the genome [2].

Whereas the short-term modulating mechanisms are conceivably essential to the immediate acclimation of the maladapted plants to the stressful condition, long-term modifications of the genome may provide the molecular basis for adaptive evolution by progenies of the stressed plants as a more adapted population to the particular condition. Indeed, it has been shown in the model plant *Arabidopsis* that various stress conditions often produce protracted effects on genome stability, leading to transgenerational changes in genome structure, and which are proposed to have been initially catalyzed by epigenetic mechanisms [3]–[5]. However, an independent study involving more kinds of stress conditions showed that stress-induced transgenerational genome instability was not a general occurrence but dependent on kinds of the stress or was simply of stochastic nature [6], thus, leaving the issue still contentious. Moreover, a recent study, also in *Arabidopsis*, demonstrated that stress-induced loss of epigenetic silencing of both a transgene and some endogenous transposable elements (TEs) were largely transitional, and showed only limited transgenerational inheritance to somatic cells of non-stressed progenies [7]. Clearly, more investigations in different plant species are needed to elucidate the generality, characteristics and extent of transgenerational heritability of epigenetic effects instigated by stressful environmental conditions on pant genomes.

Epigenetic modifications of chromatin in higher eukaryotes are essential to its functionality, as it is compromised of multiple self-reinforcing and interlaced covalent markers that are hallmarks of active or inactive chromatin states [8]. Among the known epigenetic modifications, cytosine DNA methylation prominently stands out, as it is the hitherto only well-documented epigenetic marker that is relatively stable and transgenerationally inheritable. Relative to mammalian animals, DNA methylation in higher plants is more abundant and complex, as it may occur in all sequence contexts of cytosine, including symmetric CG and CHG sites and asymmetric CHH sites [9], [10]. The addition and removal of the methyl groups to 5-cytosines of the various sequence contexts are catalyzed by distinct yet overlapping enzymes, including DNA methyltransferase 1 (*MET1*), the homologue of mammalian *DNMT1*, *CMT3* (Chromo-methyltransferase 3), a plant-specific DNA methyltransferase, and *DRM2* (domains rearranged methyltransferase), a *de novo* methyltransferase [11]–[13]. *MET1* is primarily responsible for maintenance of CG methylation [14], [15], while CMT3 is thought to maintain CHG methylation [15], [16]. *DRM2* establishes new cytosine methylation in all sequence contexts guided by small (s) RNAs and the *DRD1/polIVb* complex, using the plant-specific RNA-directed DNA methylation (RdDM) mechanism [10], [17]. While methylation of both CG and CHG sequences in plants can be faithfully perpetuated through meiosis by action of the maintenance methyltransferases, methylation of CHH most probably needs to be re-established in every generation [9]. The potentially reversible nature of DNA methylation modifications is mediated either by a passive loss of the methyl groups during DNA synthesis as a result of inefficient titration of the enzymes, or by active demethylation of previously methylated sequences by the DNA glycosylases *DME* and *ROS1* [4]. Although detailed biological functions remain to be fully elucidated, DNA methylation plays important roles in multiple fundamental cellular activities including control of gene expression, maintenance of genomic integrity, formation and perpetuation of chromatin structure, and control of genomic imprinting [16], [18], [19]. Importantly, accumulated recent evidence in both plants and animals indicates that DNA methylation is responsive to perturbation, and plays important roles in coping with both internal and environmental stress conditions [20]–[22].

Heavy metals as trace elements usually play essential biological roles for normal growth and development in plants. However, not all kinds of heavy metals are of biological importance, for example, whereas Cu^2+^ and Cr^3+^ are essential trace elements at low concentrations, Cd^2+^ and Hg^2+^ have no known functions as nutrients and seem to be toxic to plants even at very low doses [23], [24]. Thus, heavy metals, whether being essential trace elements but in excessive doses or purely toxic, may induce cellular stress responses and damage to different cellular components, e.g., membranes, proteins and DNA, and hence, constitute typical abiotic stresses to plants. Indeed, some previous studies have shown that heavy metal stress induced changes in DNA methylation [25]–[27]. However, whether the changed patterns induced by heavy metals are heritable remains largely unexplored. A recent study showed that heavy metal stress led to transgenerational alteration in the frequencies of homologous recombination (HR) and enhanced tolerance by the unstressed progenies to the same or related heavy metal treatments [28]. Although the transgenerational heritability of altered HR in this study strongly implicated an epigenetic underpinning, the exact epigenetic mechanism(s) remained to be elucidated [28].

In this study, we sought to explore whether the frequently occurring heavy metal-contaminating environmental conditions (mostly resulting from industrialization) may constitute a stress condition that affects the intrinsic DNA methylation patterns in plants; and if so, whether the epigenetic changes were transgenerationally inheritable. Furthermore, we wished to test whether the altered methylation patterns were relevant to potentially changed tolerance by progenies of the stressed plants to the particular stress condition. We addressed these questions in rice (*Oryza sativa* L.), which was chosen because its dual characteristics as a model plant for monocots and a staple food crop known to be occasionally grown in heavy metal-contaminated paddy-fields.

## Results

### Plant growth inhibition by the heavy metal treatments

**Figure 1:**
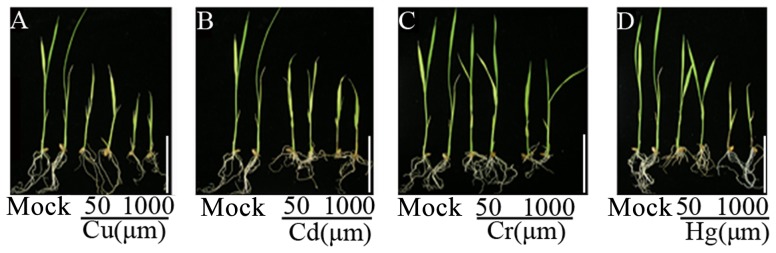
Phenotypes of rice (ssp. *japonica*, cv. Matsumae) seedlings upon heavy metal stress. The inhibitory effects were manifested by growth inhibition of shoot- and/or root-length relative to that of the mock control. Type and concentration of the used heavy metals are indicated. scale bar = 5 cm.

Treatment of 10-day old seedlings with the heavy metals, Cu^2+^, Cd^2+^, Cr^3+^ and Hg^2+^, significantly inhibited further shoot and root development of the rice (ssp. *japonica*, cv. Matsumae) seedlings (Figure 1). Moreover, the degree of growth inhibition by the treatments was clearly dose (concentration)-dependent with all four kinds of heavy-metal treatments (Figure 1). Together, these observations suggest that the heavy-metal treatments we applied represented typical abiotic stresses for the rice plants. That is, the chosen rice genotype (Matsumae) was maladapted to these adverse heavy metal conditions.

### Heavy metal stress induced locus-specific hypomethylation of both transposable elements (TEs) and protein-coding genes in somatic cells of the treated S0 generation plants

**Figure 2:**
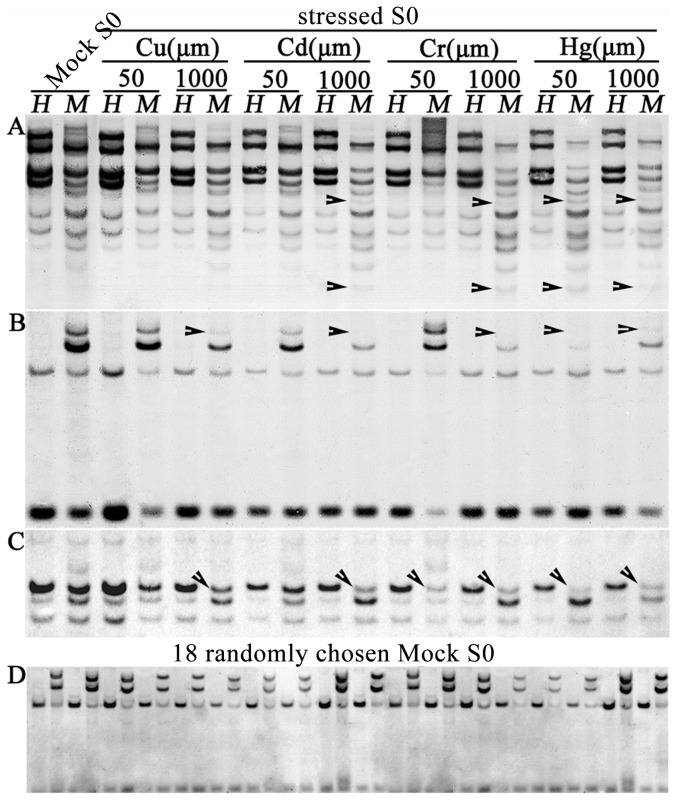
Alteration in DNA methylation patterns in the heavy metal-stressed plant seedlings (S0 generation) relative to the mock control (Mock) of rice ssp. *japonica*, cv. Matsumae. Gel-blotting patterns were generated by hybridizing the selected probes to DNA samples digested with a pair of methylation-sensitive isoschizomers, *Hpa*II and *Msp*I. Patterns of three of the 18 studied sequences (Materials and methods) are shown. Evidently, all alterations represent exclusive CHG hypomethylation (occurred only in *Msp*I digest). **A**, *Tos17*, which showed CHG hypomethylation in four of the eight treatments; **B**, *Hombox gene*, which showed CHG hypomethylation in five of the eight treatments; **C**, *CAL-11*, which showed CHG hypomethylation in six of the 8 treatments; **D**, *Hombox gene*, which showed complete stability in the methylation patterns in all 18 randomly chosen mock control plants (Mock S0). Arrowheads denote methylation alterations as a significant reduction in band signal intensity or complete band loss relative to the mock control.

We used methylation-sensitive DNA gel-blotting to detect possible alterations in DNA methylation in seedling-tissue of the heavy-metal stressed growing rice seedlings by a set of selected TEs and protein-coding genes as hybridization probes. In total, six TEs (four retrotransposons and two DNA transposons) and 12 protein-coding genes of various functional categories including those known to be involved in response to heavy-metal stress were used as hybridization probes (e.g., the P_1B_-type heavy metal ATPases-encoding genes of rice [28]). We found that compared with the mock control plants (Mock S0), changes in DNA methylation patterns occurred in seedling plants subjected to all four heavy-metal stresses (Table 1; Figure 2). Specifically, we found the following: (i) Across all 18 studied probes, one or more of the four heavy metal stresses induced DNA methylation changes in four of the six TEs (67%), and 11 of the 12 protein-encoding genes (92%) (Table 1). (ii) With regard to the two concentrations of each heavy metal treatment, usually more methylation changes occurred at the higher concentration, consistent with the dose-dependent response of growth inhibition (Figure 1). Cu^2+^ and Cr^3+^ were essential trace elements at low concentrations. Accordingly, Cu^2+^ at 50 µm did not cause any DNA methylation changes. Cr^3+^ at 50 µm however induced 11.1% DNA methylation changes (Figure 1; Table 1). When we raised the concentration by 20 times higher (1000 µm), both kinds of essential trace elements induced DNA methylation changes, and higher frequency of changes were detected in Cr^3+^ (66.7%) than in Cu^2+^ (44.4%). Cd^2+^ and Hg^2+^, being nonessential heavy metals, were toxic to plants even at very low doses, and which induced DNA methylation changes at the concentration of 50 µm (Cd^2+^: 5.6%, Hg^2+^: 66.7%). When the dose was raised to 1000 µm, Cd^2+^ induced more methylation changes (77.8%) than Hg^2+^ which remained the same as the low (50 µm) concentration (66.7%), indicating that Hg^2+^ was more toxic to the rice plants than Cd^2+^ (Figure 1; Table 1). (iii) With regard to the two cytosine residues, CG and CHG, of the 5′-CCGG sites within or adjacent to each of the probe fragments, methylation alteration occurred exclusively at the CHG sites (i.e., detectable only in *Msp*I digest) (e.g., Figure 2; Table 1). (iv) With respect to hypo- or hypermethylation changes, only hypomethylation (decrease in methylation) was detected (e.g., Figure 2; Table 1). (v) Taking the four heavy metal treatments together, from 0 to 75% methylation changes occurred for a given probe, depending on the type and concentrations of the heavy metal treatments (Table 1). (vi) With respect to penetrance of the methylation changes, a complete loss of a hybridization band signified methylation changes in all or most of the somatic cells of the seedling-tissue from all the pooled individual plants for a given treatment (e.g., Figure 2B). For other probes, the changes occurred only in portions of cells and/or only in some of the individual plants constituting a given pool (a treatment). This resulted in only a change in band intensity (relative to unaltered bands in the same lane) (e.g. Figure 2C).

**Table 1:** DNA methylation alterations on a set of transposable elements (TEs) and protein coding genes in the somatic cells (leaves) of rice ssp. japonica cv. Matsumae germinating seedlings treated with four heavy metals (S0 plants).

Probe	Genbank accession^a^	Chr^a^	Alteration in DNA methylation pattern in Matsumae treated with heavy metals (S0)^b^								
			Cu^2+^ (µm⋅L^−1^)		Cd^2+^ (µm⋅L^−1^)		Cr^3+^ (µm⋅L^−1^)		Hg^2+^ (µm⋅L^−1^)		Freq. (%)^c^
**Transposable elements (TEs)**			50	1000	50	1000	50	1000	50	1000	
*Tos17*	AC087545	10	n/n	n/n	n/n	n/-	n/n	n/-	n/-	n/-	50.0
*Osr2*	AL442110	4	n/n	n/n	n/n	n/-	n/n	n/-	n/-	n/n	37.5
*Osr36*	AP001551	1	n/n	n/n	n/n	n/-	n/n	n/-	n/-	n/n	37.5
*Osr42*	AF458768	4	n/n	n/-	n/n	n/-	n/-	n/n	n/-	n/-	62.5
*mPing*	AP005628	1–12	n/n	n/n	n/n	n/n	n/n	n/n	n/n	n/n	0.0
*Pong-sp*	AP003543	6	n/n	n/n	n/n	n/n	n/n	n/n	n/n	n/n	0.0
**Low copy protein-encoding genes**											
*Homebox gene*	AB007627	2	n/n	n/-	n/n	n/**-**	n/n	n/**-**	n/**-**	n/-	62.5
*DNA-binding protein*	X88798	5	n/n	n-	n/n	n/-	n/n	n/-	n/-	n/-	62.5
*Elongation factor*	D12821	7	n/n	n/n	n/n	n/-	n/n	n/-	n/-	n/-	50.0
*Hsp70*	X67711	11	n/n	n/n	n/n	n/n	n/n	n/n	n/n	n/-	12.5
*YF25*	DQ239435	11	n/n	n/n	n/n	n/n	n/n	n/n	n/n	n/n	0.0
*SNF-FZ14*	DQ239432	7	n/n	n/-	n/n	n/-	n/n	n/-	n/-	n/-	62.5
*S3*	AY328087	12	n/n	n/-	n/-	n/-	n/n	n/-	n/-	n/-	75.0
*CDPK-R*	AK067709	7	n/n	n/n	n/n	n/-	n/n	n/-	n/-	n/n	37.5
*CAL-2*	AK069341	7	n/n	n/-	n/n	n/-	n/n	n/-	n/-	n/-	62.5
*CAL-11*	X81393	3	n/n	n/-	n/n	n/-	n/-	n/-	n/-	n/-	75.0
*OsHMA4*	AP004184	2	n/n	n/n	n/n	n/-	n/n	n/n	n/n	n/-	25.0
*OsHMA8*	AC125472	8	n/n	n/-	n/n	n/-	n/n	n/-	n/n	n/-	50.0
Freq. (%)^d^			0.0	44.4	5.6	77.8	11.1	66.7	66.7	66.7	

**Note:**

a Determined by BlastN at NCBI;

b Changes in DNA methylation pattern is defined as: n/n: No changes in *Hpa*II and *Msp*I; n**/-**: No changes in *Hpa*II, hypomethylation in *Msp*I;

C Among 8 kinds of treatment, the frequency which can induce the alteration on DNA methylation;

d Among 18 sequences, the frequency of the specified treatment can induce the changes of DNA methylation.

To test the remote possibility that the altered methylation patterns were due to a cause other than the heavy metal treatments, or due to preexisting heterozygosity of the methylation state for the loci in question, 18 randomly selected Mock S0 plants were subjected to the same gel-blotting analysis with each of the studied probes. In all cases, monomorphic hybridization patterns denoting absence of methylation alteration or preexisting methylation heterozygosity was observed (e.g., Figure 2D). Thus, we conclude that the observed locus-specific hypomethylation in somatic cells of the treated rice seedling plants by heavy-metal resulted from the treatments.

### The modified DNA methylation patterns were transgenerationally heritable via both maternal and paternal germline transmission, and often coupled with progressive, additional modifications in the following generation

To investigate heritability of the modified DNA methylation patterns that occurred in somatic cells of the heavy metal stress treated rice seedling-plants, we selfed one individual plant derived from the Hg^2+^ (50 µm) treatment, as this treatment induced methylation modification in the majority of the studied probe sequences (66.7%) (Table 1), and the treated S0 plants survived to maturity and produced sufficient seeds for further experimentation.

**Figure 3:**
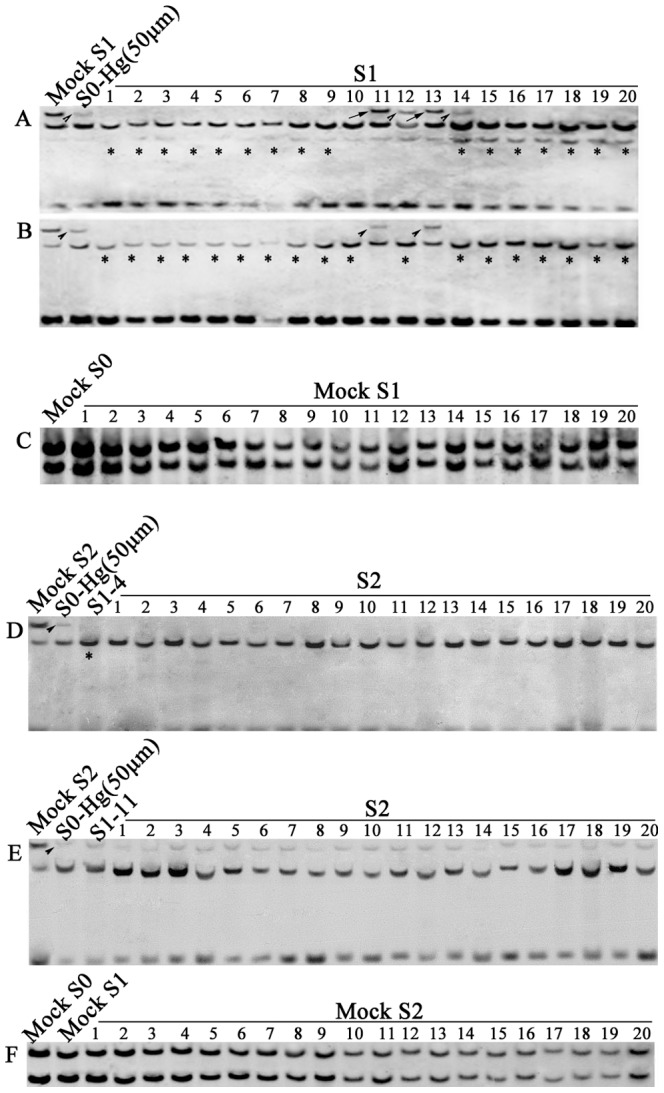
Transgenerational inheritance of the altered DNA methylation patterns induced by one (Hg^2+^) of the heavy-metal stresses at a mild concentration (50 µm) in the S0 plants (marked as S0-Hg^2+^ (50 µm) across two successive selfed generations (S1 and S2). Examples of hypomethylated patterns in the heavy-metal treated S0-generation plants, which showed “inheritance”, “new pattern” (further hypomethylation), and “reversion” to wild-type in the S1 generation (A and B), but were then stably inherited in the S2 generation (D and E). The *Hombox gene* (**A**) detected hypomethylation in S0-Hg^2+^ (50 µm) (marked with arrowhead) which in the S1 generation showed all three types of patterns, “inheritance” (plants #12 and #14, marked with arrowheads), “reversion” (plants #11 and #13, marked with arrows), and a new pattern that cannot be explained by segregation alone, and therefore most likely is associated with further methylation modification, i.e., further hypomethylation (marked with asterisks). The *DNA-binding protein* **-** *encoding gene* (**B**), plants #11 and #13 showed heritable DNA methylation patterns (marked with arrowhead). **C**, Hybridization of the *osHMA8* gene to genomic DNAs of 20 randomly chosen mock S1 plants which are selfed progenies of one wild-type control individual (Mock S0); monomorphic pattern indicate stable inheritance of intrinsic methylation patterns in the ground control plants. **D** and **E** are examples of changed methylation pattern in S2 generation (probe, *DNA-binding protein*). **D**, S2 generation showed complete inheritance of altered DNA methylation patterns in 20 randomly selected self-pollinated progenies of S1–4 (the upper band was lost in all plants, marked with asterisks); **E**, S2 generation showed complete inheritance of altered DNA methylation patterns in 20 randomly selected self-pollinated progenies of S1–11. **F**, Hybridization of the *osHMA8* to genomic DNAs of 20 randomly chosen Mock S2 plants which are selfed progenies of one Mock S1 individual; monomorphic patterns indicate stable inheritance of the default methylation pattern of wild-type control plants.

We performed methylation-sensitive gel-blotting analysis for eight sequences (Table 2) which detected conspicuous methylation modifications in the S0 plants (Table 1). We found three types of methylation patterns occurred among the 20 randomly chosen S1 plants descended from a single S0 plant treated with Hg^2+^ (50 µm): “inheritance” of the modified patterns of the parental S0 plant, “new patterns” superimposed on the modified patterns of the S0 plant, and “reversion” to the original patterns of the untreated control plants (e.g., Figures 3A and B; Table 2). Each type of the methylation patterns occurred at variable frequencies among the eight probes, but together, the type of “new pattern” was the predominant type that occurred at frequencies from 45% to 95% depending on the probes (Table 2). The other two types of patterns, “inheritance” and “reversion”, together made up the rest frequencies (5% to 55%). These three patterns across the 20 S1 plants could, at least in part, be explained by Mendelian segregation of modified methylation patterns in the parental S0 plant if the loci in question were heterozygous, i.e., harboring different methylation epialleles in the two homologous chromosomes (e.g., Figures 2A and B). Nonetheless, the number of the S1 plants showing new patterns greatly exceeded the ratio expected according to Mendelian segregation (Table 2). Moreover, the new patterns were often characterized with appearance of additional new bands that were superimposed on the segregated patterns (e.g., Figure 2 A and B). Thus, further DNA methylation modifications in the same direction (i.e., hypomethylation) occurred in the S1 generation. Notably, as in the S0 plants, the additional hypomethylation occurred exclusively at the CHG sites (e.g., Figures 2A and B; Table 1). It implies the same eliciting signal(s) to be responsible in the two consecutive generations (e.g., Figures 3A and B; Table 2).

**Table 2:** Transgenerational alteration and inheritance of DNA methylation patterns in 20 randomly chosen S1 plants derived from a Hg^2+^(50 µm)-stressed S0 individual.

Probe	Alteration of DNA methylation pattern in the S0 plant and its S1 progenies																					Type and Freq. (%) of pattern		
	S0-Hg^2+^ (50 µm)	1	2	3	4	5	6	7	8	9	10	11	12	13	14	15	16	17	18	19	20	Inherit. of S0 pattern	Rev. to Mock pattern	New pattern
*Tos17*	-	–	–	–	**r**	–	–	–	**i**	**i**	**i**	**r**	**r**	**r**	–	–	**i**	**r**	**–**	**r**	**r**	20.0	35.0	45.0
*Hombox gene*	-	–	–	–	–	–	–	–	–	–	–	**r**	**i**	**r**	**i**	–	–	–	–	–	–	10.0	10.0	80.0
*DNA-binding protein*	-	–	–	–	–	–	–	–	–	–	–	**i**	–	**i**	–	–	–	–	–	–	–	10.0	0.0	90.0
*SNF-FZ14*	-	–	–	–	–	–	–	–	–	–	–	**i**	–	**r**	–	–	–	–	–	–	–	5.0	5.0	90.0
*S3*	-	–	–	–	–	–	–	–	–	–	–	–	–	**r**	–	–	–	–	–	–	–	0.0	5.0	95.0
*CAL-11*	-	–	–	–	**–**	**i**	**i**	**i**	–	–	–	**i**	**i**	**i**	**i**	**i**	**i**	**i**	–	–	–	50.0	0.0	50.0
*OsHMA4*	**n**	-	-	-	-	-	-	-	**n**	**n**	**n**	**n**	**n**	**n**	**n**	**n**	**n**	**n**	**-**	**n**	-	na	na	45.0
*OsHMA8*	**n**	-	-	-	-	-	-	-	-	-	-	**n**	-	**n**	-	-	-	-	-	-	-	na	na	90.0

**Explanation of symbols:** - denotes hypomethylation changes in the S0 plant or its S1 progeny plants; n denotes no methylation change in the S0 plant or its S1 progeny plants; – denotes S1 plants showing further decrease in DNA methylation; r denotes S1 plants being reverted back to the Mock control patterns; i denotes S1 plants having inherited the modified patterns of S0 plant; na denotes not applicable to the particular case.

We also performed the same gel-blotting analysis for an identical number (20) of randomly chosen, selfed progeny plants (Mock S1) derived from one control plant (Mock S0), and we observed only monomorphic patterns (e.g., Figure 3C), indicating transgenerational stability of the original “default” methylation patterns under unstressed, normal growing conditions.

Next, we selected two S1 plants, #4, which predominantly showed new pattern (further hypomethylation), and #11, which showed all three types of patterns (new, inheritance and reversion — depending on probes) to produce their S2 progenies. We performed the same methylation-sensitive gel-blotting analysis for four probes in 20 randomly chosen S2 plants from each of the two S1 plants. We found that the new patterns which had been detected in S1 were stably inherited to their S2 progenies with 100% frequency, indicating all the loci in question being homozygous with regard to the methylation epialleles, and no further modification had occurred in the S2 generation (e.g., Figures 3D and E; Table 3). In contrast, for one modification (probe *OsHMA8*) that occurred in the S1 plant (but not in S0), a frequency of 25% was observed for inheritance of the modified pattern (Table 3), indicating that this specific locus was heterozygous for the methylation state.

**Table 3:** Transgenerational alteration and inheritance of DNA methylation patterns in 20 randomly chosen S2 plants derived from two S1 individual plants, S1(#4) and S1(#11) which were derived from a single Hg^2+^ (50 µm)-stressed S0 individual.

Probe			DNA methylation patterns in S2 plant individuals																				Frequency (%)		
	S0-Hg^2+^ (50 µm)	S1	1	2	3	4	5	6	7	8	9	10	11	12	13	14	15	16	17	18	19	20	Inherit. of S1 pattern	Rev. to S0 pattern	New pattern
*DNA-binding protein*	-	(#4) –	–	–	–	–	–	–	–	–	–	–	–	–	–	–	–	–	–	–	–	–	100	0	0
		(#11) -	-	-	-	-	-	-	-	-	-	-	-	-	-	-	-	-	-	-	-	-	na	0	0
*CAL-11*	-	(#4) –	–	–	–	–	–	–	–	–	–	–	–	–	–	–	–	–	–	–	–	–	100	0	0
		(#11) –	–	–	–	–	–	–	–	–	–	–	–	–	–	–	–	–	–	–	–	–	100	0	0
*OsHMA4*	n	(#4) -	-	-	-	-	-	-	-	-	-	-	-	-	-	-	-	-	-	-	-	-	100	0	0
		(#11) n	n	n	n	n	n	n	n	n	n	n	n	n	n	n	n	n	n	n	n	n	na	na	na
*OsHMA8*	n	(#4) -	-	r	r	-	r	r	-	r	r	r	r	r	r	r	-	r	-	r	r	r	25.0	75.0	0
		(#11) n	n	n	n	n	n	n	n	n	n	n	n	n	n	n	n	n	n	n	n	n	na	na	na

**Explanation of symbols:** - denotes hypomethylation changes in the S0 plant or its S1 progeny plants; n denotes no methylation change in the S0 plant or its S1 progeny plants; – denotes S1 plants showing further decrease in DNA methylation; r denotes S1 plants being reverted back to the Mock control patterns; i denotes S1 plants having inherited the modified patterns of S0 plant; na denotes not applicable to the particular case.

We performed the same gel-blotting analysis with the same eight probes in an identical number (20) of selfed progeny plants (Mock S2) derived from Mock S1, and again we observed only monomorphic patterns, further indicating transgenerational stability of the original, “default” methylation patterns (e.g., Figure 3F).

We further explored heritability to the S3 generation of the modified methylation patterns that appeared stabilized in the S2 generation. We investigated 20 randomly chosen S3 plants derived from a single S2 plant (#17) with four probes (*DNA*-*binding protein*, *CAL-11*, *osHMA4* and *osHMA8*) as representatives. We found that each of the probes showed 100% inheritance to the S3 plants for the modified methylation patterns that originally occurred at S0 (data not shown). This suggests that the heavy metal stress-induced DNA methylation modifications after becoming homozygous for the epiallelic state can be stably inherited thereafter under unstressed normal conditions.

To test whether there exists a bias with regard to the maternal *vs.* paternal germinal transmission of the modified DNA methylation patterns, we generated a pair of reciprocal F1 hybrids between an unstressed wild-type control plant and a S2 individual plant (S2#7) derived from S1#4 (Figure 3D) that had originated from a Hg^2+^ (50 µm)-treated S0 plant. Remarkably, twenty randomly chosen S3 progenies from S2#7 analyzed for four representative probes (described above), showed no segregation. Thus, it was assumed that at least for these four representative loci, the plants were homozygous for the modified methylation patterns. We analyzed 20 F1 plants of each of the crossing directions by gel-blotting analysis using the same four representative probes. We found variable frequencies (from 47.5 to 51.3%) of inheritance of the modified patterns in the backcrossed F1 plants depending on the probes, but collectively, the mean frequencies for maternal and paternal germinal transmissions across the four probes were statistically insignificant based on a Chi-square test (Table 4).

**Table 4:** Maternal vs. paternal germinal transmission of the modified DNA methylation patterns in a pair of reciprocal F1 hybrids (each crossing direction containing 20 individuals) of an unstressed wild-type control plant and a S2 individual plant (S2#7) derived from S1#4 that had originated from a Hg^2+^ (50 µm)-treated S0 plant.

“Maternal×paternal” cross	probe	Transmission frequency (%) in 20 F1 hybrid individuals
S2#7×wild-type control		
	*DNA-binding protein*	35
	*CAL-11*	55
	*OsHMA4*	20
	*OsHMA8*	80
**Mean frequency (%)**		**47.5**
Chi-square value		0.4
Wild-type control×S2#7		
	*DNA-binding protein*	70
	*CAL-11*	25
	*OsHMA4*	75
	*OsHMA8*	35
**Mean frequency (%)**		**51.3**
Chi-square value		0.38

**Note:** Compared with the Chi-square values, the *P* values of he mean frequencies greatly exceeded 0.05 indicating that there was no significant difference between maternal and paternal germinal transmission of modified DNA methylation patterns for the four studied probe.

### The modified DNA methylation patterns were correlated with altered expression state of genes involved in chromatin-regulation

**Figure 4:**
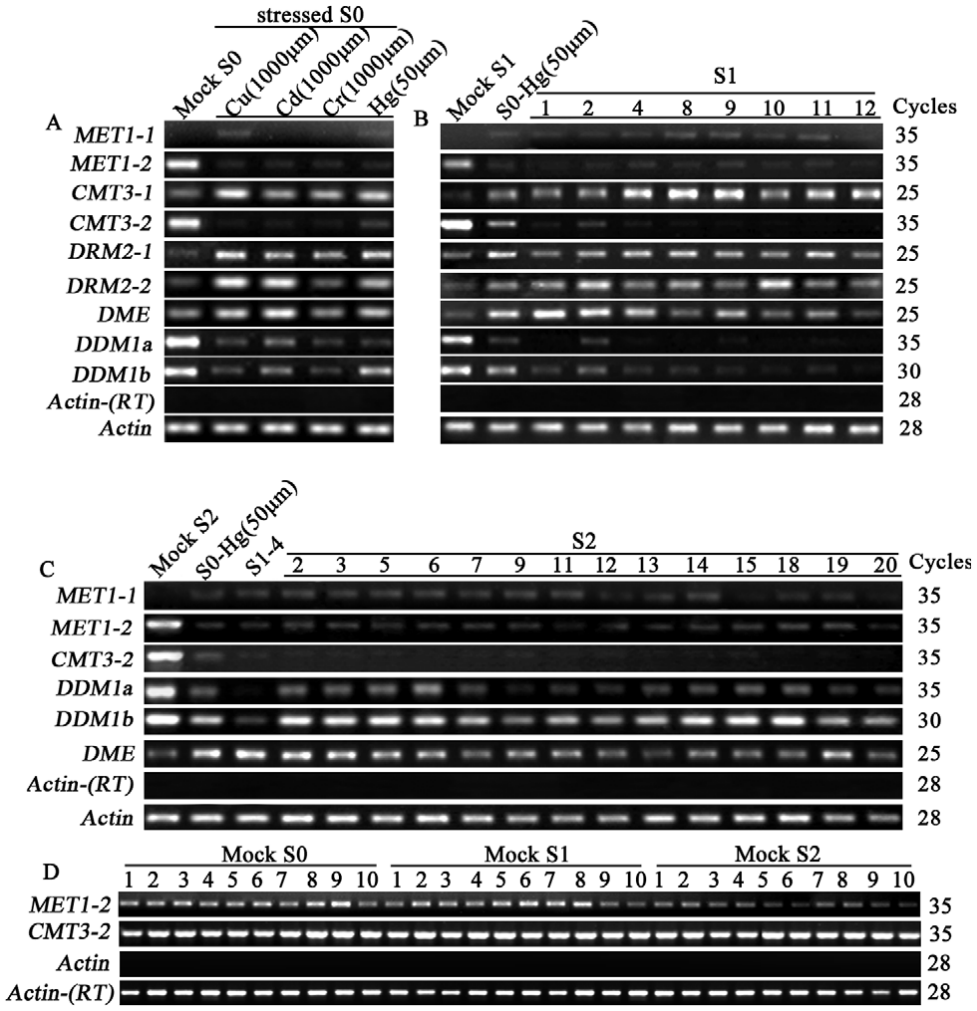
Alteration in the steady-state transcript abundance of a set of nine chromatin-related genes encoding for putative DNA methyltransferases (six), 5-methylcytosine DNA glycosylase (one) and the *SWI/SNF* chromatin remodeler (*DDM1*) (two) in the heavy-metal treated rice plants at the S0, S1 and S2 generations determined by semi-quantitative RT-PCR analysis. (**A**) Transcript abundance of the nine genes in the Mock S0 and each of the four kinds of heavy-metal treatments at the indicated concentration labeled as stressed S0. (**B**) Transmission of the altered transcript abundance as well as further alteration of each of the nine genes by heavy-metal Hg^2+^ (50 µm) treatment from S0 to its eight S1 progenies. (**C**) Transmission of the altered transcript abundance of six genes in one S1 individual (S1#4) to its 14 S2 progenies. (**D**) Two examples showing lack of fluctuation for the chromatin-related genes in the untreated mock control plants across generations. For all analysis, three batches of independent RNAs were isolated from seedling-leaf tissue at the same stage, and RT-PCR was performed with each of them. The results were highly reproducible among the three independent RNA batches, and hence, only one experiment was presented. Gene name and amplification cycles are labeled. The rice *Actin* gene (Genbank accession number:X79378 ) was used as an internal control in all cases. Lack of genomic DNA was validated by the *Actin* gene on template without RT.

To explore the possibility that the transgenerationally heritable modifications of DNA methylation subsequent to the heavy-metal treatments might be related to persistent perturbation of the expression state of chromatin-related genes, we measured the mRNA steady-state abundance in selected plants of the S0, S1 and S2 generations for a set of nine chromatin-state related genes encoding for putative DNA methyltransferases (*MET1-1*, *MET1-2*, *CMT3-1*, *CMT3-2*, *DRM2-1* and *DRM2-2*), a 5-methylcytosine DNA glycosylase (*DME*) and the *SWI/SNF* chromatin remodeler (*DDM1*) (*DDM1a* and *DDM1b*) by semi-quantitative reverse transcription-PCR (RT-PCR) analysis (Figure 4). The S0 plants were chosen according to the gel-blotting results for those that showed the most conspicuous modifications in DNA methylation pattern, which included the heavy-metal treatments of Cu^2+^ (1000 µm), Cd^2+^ (100 µm), Cr^3+^ (1000 µm) and Hg^2+^ (50 µm). For the S1 and S2 progenies, only those of the Hg^2+^ (50 µm) treatment were studied, and the individual plants were selected to represent the three patterns of DNA methylation modifications i.e. inheritance, new pattern and reversion to wild-type pattern (Figure 3).

For the directly treated S0 plants, the following observations were made based on RT-PCR profiles of the nine genes: (1) one gene (*MET1-1*) was intrinsically not expressed in leaf-tissue of the control plants (Mock S0) but was activated to express (though at a low level) in the S0 plants treated with Cu^2+^ (1000 µm) and Hg^2+^ (50 µm) (Figure 4A); (2) four genes (*MET1-2*, *CMT3-2*, *DDM1a* and *DDM1b*) were highly expressed in control plants, but were silenced or significantly down-regulated in the S0 plants of all four heavy-metal treatments (Figure 4A); (3) four genes (*CMT3-1*, *DRM2-1*, *DRM2-2* and *DME*) were moderately expressed in control plants, but were significantly up-regulated in the S0 plants of all four heavy-metal treatments (Figure 4A).

For the S1 generation, we measured the steady-state mRNAs of these nine chromatin-state related genes on eight individuals. The data (Figure 4B) showed that (1) two genes (*MET1-1* and *MET1-2*), which were respectively activated and silenced in the S0 plants, retained the altered expression of their S0 parents in all S1 individuals; (2) three genes (*CMT3-2*, *DDM1a* and *DDM1b*), which were down-regulated in S0, showed in the S1 progenies a tendency of further down-regulation in all or most of the studied S1 plants; (3) the last four genes (*CMT3-1*, *DRM2-1*, *DRM2-2* and *DME*), which were up-regulated in S0, displayed in the S1 progeny individuals a tendency of either stable inheritance or further enhanced up-regulation.

Next, we selected 14 S2 individuals derived from one S1 individual (S1#4) to further test inheritance or variation of the altered expression states of six genes (*MET1-1*, *MET1-2*, *CMT3-2*, *DDM1a*, *DDM1b* and *DME*) that showed the most clear-cut difference in steady-state transcript abundance between the heavy metal treated and untreated mock plants in both S0 and S1 generations (Figures 4A and B). We found that that four genes (*MET1-1*, *MET1-2*, *CMT3-2* and *DME*) showed stable inheritance of the expression sates of their S1 parent, while two genes (*DDM1a* and *DDM1b*) showed largely reversion gene expression to those of the mock plants (Figure 4C).

Finally, to test the remote possibility that the expression states of these chromatin-regulation related genes were inherently fluctuating even in unstressed mock control plants of the rice cultivar used (Matsumae), we assessed the expression of these genes on 30 randomly chosen individuals from three consecutive selfed generations (Mock S0, Mock S1 and Mock S2). Similar to the situation for stability of DNA methylation pattern in the mock control plants (Figures 2 and 3), the inter-individual expression levels for each of these nine studied genes was remarkably consistent (e.g., Figure 4D).

### Progenies of the heavy metal-stressed plants showed enhanced tolerance to the same stress

**Figure 5:**
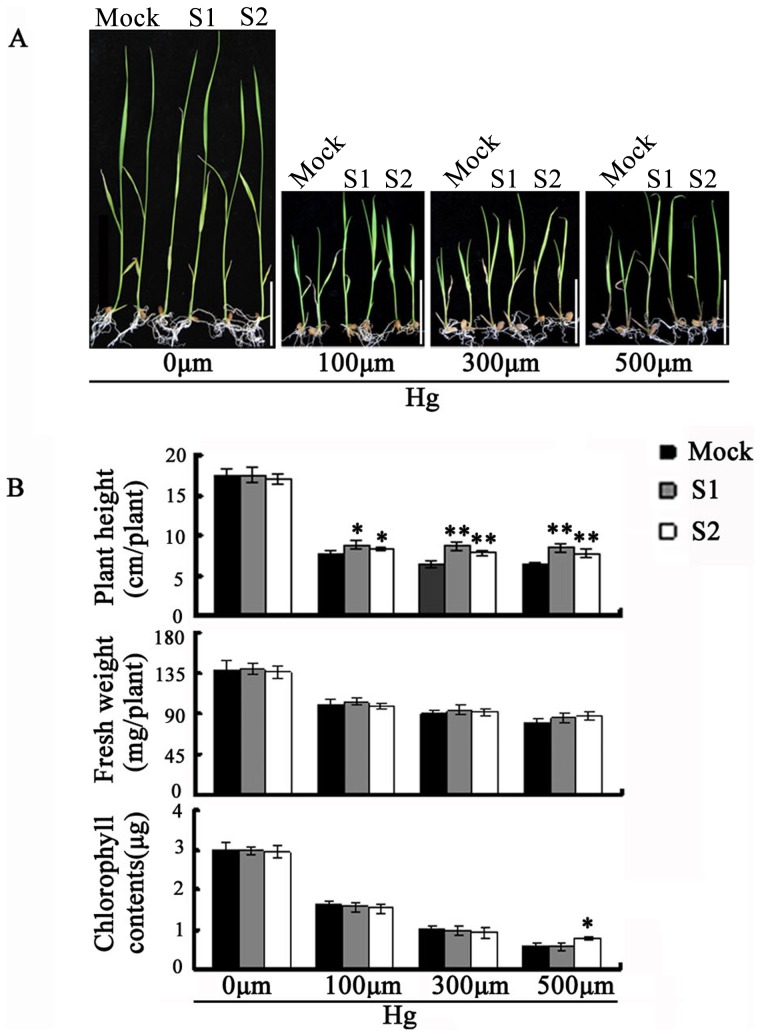
Phenotypes and quantitative measurements of traits from mock control and progenies of heavy metal (Hg^2+^ at 100 µm) stressed plants under normal and heavy metal stress conditions (Hg^2+^ at 100, 300 and 500 µm, respectively). (**A**) The overall seedling phenotypes of plants (two individuals are shown) under normal (left-most) and three heavy metal (from left to right: 100, 300 and 500 µm) Hg^2+^ stress conditions, Bars = 5 cm. (**B**) Phenotypic analysis of mock control plants (Mock) and two successive selfed-pollinated progenies, S1 and S2, derived from S0-Hg^2+^ (50 µm). Seeds were germinated and seedlings grown for 10 days on Hoagland nutrient solution with or without the indicated concentrations of Hg^2+^. Plant height, fresh weight and chlorophyll content were measured. For all measurements, data are from three independent experiments each containing 10 individual plants. Error bars represent standard error (SE). * and ** denote statistical significance at 0.05 and 0.01 levels respectively in student's test.

To test whether progenies of the heavy metal-stressed plants would show the same or altered sensitivities to the same stress condition, we subjected S1 and S2 plants derived from a single S0 individual stressed with Hg^2+^ (50 µm) to Hg^2+^ treatments with three concentrations (100, 300 and 500 µm) together with mock control plants. After growing for 10 days, we scored three traits (plant height, fresh weight, and chlorophyll content) that have been commonly used as measurements of heavy metal sensitivity [29]. Data showed that whereas at the unstressed normal condition, no difference was noted between progenies (S1/S2) of the Hg^2+^-stressed and mock control plants, there were significant differences between the S1/S2 progeny and the mock control plants at all three concentrations of Hg^2+^ treatments. The S1 and S2 progeny plants showed clearly enhanced tolerance to the heavy metal stress (Figure 5). The most significant difference was manifested by plant height in comparison of S1/S2 progeny plants and their mock control plants at all three Hg^2+^ concentrations; the chlorophyll content was significantly higher only at concentration 500 µm in the S2 plants, while no significant difference was detected in comparisons for fresh weight (Figure 5). Together, these results demonstrated that progenies derived from a heavy metal (Hg^2+^)-stressed rice plant “remembered” the stress condition their parent experienced, and developed heritable, enhanced tolerance to the stress condition.

## Discussion

Although accumulated empirical evidence in diverse organismal taxa has established DNA methylation to be the most stable epigenetic marker, it is metastable compared to the primary nucleotide sequence under various internal and external stress conditions. This inherit epigenetic flexibility may contribute to the organism's immediate response and long-term evolvability towards adaption to changing environmental conditions [26], [30], [31]. Our data showed that heavy metal stress represents a condition under which the cytosine methylation patterns of both TEs and protein-coding genes can be significantly altered in rice. A salient feature of the methylation alterations induced by this particular stress is that all detected alterations at the resolution of gel-blotting are hypomethylation of the CHG sites, whereas the CG methylation remains unaffected. Although this is in accordance with the idea that plant CHG methylation is more prone to perturbation by environmental stresses than CG methylation [4], the exclusiveness of our observation is striking and unprecedented in a wild-type (non-mutant) plant, and pointing to a need to elucidate the induction signal(s) and the underlying mechanisms.

The cause for specific CHG hypomethylation as a result of heavy metal stress remains unknown. Nonetheless, we note that the changes are concomitant with differentially altered steady-state transcript levels of the chromatin-related genes known to be responsible for faithful maintenance of the various methylation patterns in plants. For example, simultaneous down-regulation of the *DDM1* and *CMT3* genes and up-regulation of the *MET1*, *DRM* and *DME* genes are consistent with reinforced stabilization for CG methylation but compromised maintenance for, and/or active removal of, CHG methylation [4], [10], [22], [32]–[34]. Thus, it appears possible that disruption of the otherwise coordinated expression of these chromatin-regulation genes subsequent to the heavy metal stress is a major cause for changes in the specific DNA methylation patterns. Alternatively, it is also possible that the loss of 5-methyl-cytosines is independent of activity and/or titration of these enzymes, but a byproduct-effect of the heavy metals on DNA *per se*. For example, both biotic and abiotic stresses (including heavy metals) are known to cause an increased production of reactive oxygen species (ROS) [35]. ROS possesses endonuclease activities and hence may cause DNA damages including double-stranded breaks (DSBs), which may interfere with capability of DNA as an acceptor for the methyl groups [36], and resulting in passive loss of methylation at the affected sites. This later scenario however cannot explain the remarkable specificity of loss of CHG methylation only.

In recent years, the concept of transgenerational epigenetic inheritance [37], [38] has drawn much attention in the scientific community because it mirrors the theory of inheritance of acquired traits originally put forward by the eminent evolutionary biologist Jean-Baptiste Lamarck in 1809. This theory of Lamarck was largely ignored for many decades mainly because lack of a mechanistic basis. With the rapid advancement of epigenetics, Lamarck's idea has been experiencing resurgence. Although this idea remains contentious in multicellular animals [39], it is more readily acceptable in plants due to fundamental differences between the two organismal kingdoms with respect to presence *vs.* absence of epigenetic “erasure and resetting” dynamics across ontogeny, and the earlier sequestering of germline in animals *vs.* lack thereof in plants [37]. However, even in plants, discrepant results regarding the extent of transgenerational epigenetic inheritance were observed by different studies, particularly in regard to heritability of stress-induced epigenetic modifications. For example, Molinier *et al.* [3] found that in *Arabidopsis thaliana* plants treated with short wave-length radiation (ultraviolet-C) or flagellin (an elicitor of plant defense), the frequencies of somatic homologous recombination (SHR) of a reporter transgene was significantly increased, and this trait of enhanced homologous recombination is epigenetic in nature and persisted in unstressed subsequent generations [3]. Whereas this study provided a striking example of epigenetic-based transgenerational stress memory in plants, a follow-up study by Pecinka *et al.* [6] using more physical and chemical stress treatments on various *Arabidopsis thaliana* lines demonstrated that although the treatments caused significantly increased levels of SHR, the acquired trait of SHR showed only limited and stochastic inheritance to subsequent generations, suggesting that the epigenetic mechanism-mediated transgenerational stress memory is not a general response in *Arabidopsis* [6]. This later conclusion was corroborated by another study, also in *Arabidopsis*, which showed that stress-induced loss of epigenetic silencing of both a transgene and some endogenous transposable elements (TEs) were largely transitional and confined to the stressed generation [7].

We show in this study that heavy metal stress caused extensive hypomethylation in somatic cells (leaves) of the directly treated rice plants (S0). In selfed progenies (S1) of the treated plants, three types of methylation patterns were observed: “inheritance” of the modified patterns of the parental S0 plant, “new pattern” superimposed on the modified patterns of the S0 plant, and “reversion” to the original patterns of the wild-type control plants. The occurrence of “new pattern” cannot be explained by Mendelian inheritance; instead, it suggests that the same “induction signal” probably had been inherited to the S1 generation and continued to exert its effect. A similar observation was made in salt (NaCl)-stressed *Arabidopsis thaliana* plants, which showed higher homologous recombination rates, higher expression of AtRad51, lower expression of AtKu70 in the progenies of the exposed plants than their parents [40]. This across-generation “transfer of substance” has been reported previously in animals, and may include parental nutrient provision and signaling molecules [39] but cannot be considered *bona fide* transgenerational inheritance [38]. Nonetheless, our finding that all the modified patterns that occurred in S0 and a great majority of the further modified patterns (new patterns) that occurred in S1 were stably transmitted to at least two additional successive generations, S2 and S3, provides unequivocal evidence for transgenerational inheritance of the modified DNA methylation patterns. In addition, we demonstrate that no bias existed for maternal *vs.* paternal transmission of altered methylation patterns between a pair of reciprocal F1 hybrids of a S2 plant with modified methylation pattern and a wild-type plant. Together, our results clearly show that heavy metal-induced DNA methylation changes in rice are readily inheritable to organismal generations, and hence, in support of transgenerational epigenetic inheritance in plants [41]–[43]. Thus, environmental induction of heritable modifications in DNA methylation may provide a plausible molecular underpinning for the Larmarkin inheritance of acquired traits.

It has been proposed that CG methylation in *Arabidopsis* provides the scaffold for transgenerational epigenetic inheritance, and therefore stable transmission is often related to altered DNA methylation in a CG context [44]. However, two recent studies showed that exposure of *Arabidopsis* to bacterial speck disease caused transgenerational systemic acquired resistance (SAR), which was mediated via CHG hypomethylation [45], [46]. These findings showed for the first time that DNA methylation changes in CHG context have important functions in transgenerational inheritance of acquired disease resistance in plants. Remarkably, our results also show that in rice the heavy metal-induced CHG hypomethylation changes can be stably transmitted without entailing an alteration of CG methylation, suggesting a conserved mechanism might exist in rice and *Arabidopsis* with regard to epigenetic inheritance of CHG methylation and its biological significance, which clearly warrants further investigations.

Several studies in plants have suggested that specific stressful environmental conditions experienced by the progenitor led to enhanced tolerance to the same or related conditions in the progenies [42], [47], [48], a phenomenon known as “transgenerational hardening” (reviewed by [28]). There have also been numerous demonstrations of stress-induced epigenetic variation and inheritance particularly in plants. However, as pointed out [44], few studies have shown an intrinsic link between the two phenomena. We show in this study that the heavy metal-induced heritable CHG hypomethylation occurred concomitantly with enhanced tolerance to the same heavy metal by progenies derived from the stressed progenitor plants, suggesting that the two phenomena might be causally linked to each other. In addition, some studies have indicated that a mild stress might induce more transgenerational changes and stress tolerance than a severe stress [6], [20]. In this study, we also found that when the parental rice plants were treated with a mild heavy metal stress concentration (50 µm), their selfed progenies (S1 and S2) showed enhanced tolerance to heavy metals not only to the same concentration (50 µm) but also to higher concentrations (100, 300 and 500 µm) compared with the mock control plants whose progenitor did not experience the stress. This transgenerational adaptation indicates that plants may indeed “memorize” an adverse environmental condition and modify their progenies' epigenome to prepare for and better fit to the adverse condition(s).

## Methods

### Plant material

Rice (*Oryza sativa* L.) ssp. *japonica*, cv. ‘Matsumae’ was used throughout this study.

### Heavy metal treatments

Seeds were washed thoroughly with distilled water and then germinated in the dark in Petri dishes containing distilled water at 28°C. After a 2-day incubation, germinated seeds were transferred to a greenhouse at 26°C under 12 h/12 h light/dark regime. Ten day-old, seedling plants were subjected to different heavy metals at variable concentrations: Cu^2+^ (50, 1000 µm⋅L^−1^ supported by CuSO_4_); Cd^2+^ (50, 1000 µm⋅L^−1^ supported by CdCl_2_); Cr^3+^ (50, 100 µm⋅L^−1^ supported by CrCl_3_); Hg^2+^ (50, 1000 µm⋅L^−1^ supported by HgCl_2_) in the Hoagland nutrient solution for 7-day. Mock controls were grown simultaneously in the Hoagland nutrient solution. Seedling plants were transplanted to normal paddy field. Leaves were harvested at appropriate time points, immediately frozen in liquid nitrogen and stored at −80° until use. The plants were marked as “stressed S0” for heavy metal treated plants and “Mock S0” for mock control plants. Panicles of several selected stressed S0 and Mock S0 plants were bagged fro self-pollination and seeds were collected to produce the next generation plants, S1 and Mock S1, respectively. In the same way, S2 and S3 plants from the heavy metal treated and mock control were produced.

### Methylation-sensitive DNA gel-blotting

Genomic DNA was isolated from expanded leaves of heavy metal-stressed and mock control rice plants by a modified CTAB method [49] and purified by phenol extractions. For methylation analysis, the DNA (5 ug per sample) was digested by a pair of methylation-sensitive isoschizomers, *Hpa*II and *Msp*I. Digested DNA was run through 1% agarose gels and transferred onto Hybond N^+^ nylon membranes (Amersham Pharmacia Biotech, Piscataway, New Jersey) by the alkaline transfer recommended by the supplier. For probes, specific primers for a set of 12 protein-coding genes and six mobile elements (TEs) were designed based on sequences deposited at Genbank. (Table S1), and the delineated fragments were amplified by PCR amplification at an annealing temperature of 58–60°C. Authenticity of the PCR products was verified by sequencing. The fragments were gel-purified and labeled with fluorescein-11-dUTP by the Gene Images random prime-labeling module (Amersham Pharmacia Biotech). Hybridization signal was detected by the Gene Images CD2+P-Star detection module (Amersham Pharmacia Biotech) after washing at a stringency of 0.2×SSC, 0.1% SDS for 2×50 min. The filters were exposed to x-ray films.

### Reverse-transcription (RT-PCR) analysis

The protocol was essentially as reported [50]. Specifically, total RNA was isolated from expanded young leaves at the same developmental stage as that used for DNA isolation by the Trizol Reagent (Invitrogen), following the manufacturer's protocol. The RNA was treated with DNaseI (Invitrogen), reverse-transcribed by the SuperScriptTM RNase H-Reverse Transcriptase (Invitrogen), and subjected to RT-PCR analysis using gene-specific primers. A rice *Actin* gene (Genbank accession X79378) was used as a control for normalization of RNA input. The primers for all studied sequences including a set of sequences encoding for putative DNA methyltransferases (six), 5-methylcytosine DNA glycosylases (one), the *SWI/SNF* chromatin remodeler (two) were designed by the Primer 3 program (http://www.frodo.wi.mit.edu/cgi-bin/primer3/primer3_www.cgi) and are listed in Table S1. DNA contamination was tested by inclusion of RNAs without RT. Different cycles were used for the various studied genes, which ensured that the amplifications were within the linear range for each gene. Three batches of independently prepared total RNAs were used as technical replications. The amplicons were visualized by ethidium bromide staining after electrophoresis through 2% agarose gels.

### Measurements of Phenotypes

Seeds of the mock control plants and S1 and S2 progeny plants derived from a single S0 individual stressed with Hg^2+^ (50 µm) were germinated and their seedlings grew for 10 days, then they were treated with different concentrations of Hg^2+^ (0 µm⋅L^−1^, 100 µm⋅L^−1^, 300 µm⋅L^−1^ and 500 µm⋅L^−1^), as described above. Plants were collected and their weights and shoot lengths were measured. To assess their chlorophyll content, leaves were harvested and chlorophyll was extracted with 10 mL 95% ethanol from 0.2 g samples. An aliquot of the extracts was taken to measure A645 and A663 with a spectrophotometer. Specific chlorophyll content was determined as reported [51].

## Supporting Information

Table S1 **Database information and sequence-specific primers used for Southern blotting probe amplification (from genomic DNA) and/or for RT-PCR analysis.**
(DOC)
