# Supplementary material for: Transgenerational Inheritance of Modified DNA Methylation Patterns and Enhanced Tolerance Induced by Heavy Metal Stress in Rice (Oryza sativa L.)
Source: PLoS One. 2012 Sep 11;7(9):e41143. doi: 10.1371/journal.pone.0041143 (PMC3439459; doi:10.1371/journal.pone.0041143)
Supplement: Table S1 — Database information and sequence-specific primers used for Southern blotting probe amplification (from genomic DNA) and/or for RT-PCR analysis. (DOC) [file pone.0041143.s001.doc]

**Tables S1** Database information and sequence-specific primers used for Southern blotting probe amplification (from genomic DNA) and/or for RT-PCR analysis

| Gene name | Genbank accession | Forward primer (5’-3’) | Reverse primer (5’-3’) |
| --- | --- | --- | --- |
| *OsActin* | X79378 | cgtgtgcgataatggaactg | tctgggtcatcttctcacga |
| *Tos17* | AC087545 | aaagggaaactcagcgaaca | gagggcacatagtggagagc |
| *Osr2* | AL442110 | cacaccagcaccaagtccta | tcgatcgctttaggttgctt |
| *Osr36* | AP001551 | ccctgaatccaccaagaaaa | ggcagtctcgagaaggtgac |
| *Osr42* | AF458768 | ccacagatcatcatttctgacc | ccccttgaagactgacttgc |
| *mPing* | AP005628 | gtcacaatgggggtttcact | ggccagtcacaatggctagt |
| *Pong-sp* | AP003543 | ggggtgaaacagcattgaga | tgtggttgcaaagaagacc |
| *Hombox gene* | AB007627 | ttgatggaaatgatgggtca | actgcatcgtgcatcaaaac |
| *DNA-binding protein* | X88798 | agaatgccactcctcctgtg | gtcctcccttctgtgctgag |
| *Elongation factor* | D12821 | acctctccggcaagacctac | ttacaagccgctctgcagtt |
| *Hsp70* | X67711 | cccatcttggtggtgaagat | gtcctcagcagacacgttca |
| *YF25* | DQ239435 | ccggatggaagaagagatca | tggagctcaaggcgatattt |
| *SNF-FZ14* | DQ239432 | tgaggctgtcagcatgatct | atctgaggatgttgcgcttc |
| *S3* | AY328087 | ggtgcactgcttcttcattg | tcgagttgtcctcgtcctct |
| *CDPK-R* | AK067709 | CAGACATGGGGTGAGGTTTT | CCACCATAGAGCCCTGACAT |
| *CAL-2* | AK069341 | AAACTGTCCCCTTCCGAGTT | CGCTTCCTCCTCGTAAAG |
| *CAL-11* | X81393 | ATGGACTCCCAATCAACTCG | CTCCATGACCCCAGTGAGAT |
| *OsHMA4* | AP004184 | tgctggggaaatatctggag | cttctgaactggagcccttg |
| *OsHMA8* | AC125472 | ataatggaggaggccgaact | cctgccttttcatgtggagt |
| *OsMET1-1* | AF462029 | ttggagctcatccctatgaaagc | aagggtgatgatactaacagctagc |
| *OsMET1-2* | BK001405 | tattgcacggaaaggagc | gctggctgtggaggttta |
| *OsCMT3-1* | [AK109728](http://www.tigr.org/tigr-scripts/osa1_web/gbrowse/rice/?name=LOC_Os05g13790) | ggctacaaccgcaataga | accagaggacgaccagat |
| *OsCMT3-2* | [AK112062](http://www.tigr.org/tigr-scripts/osa1_web/gbrowse/rice/?name=LOC_Os10g01570) | gcttagccgtaaagaaatgc | ccaacagggataccaacaga |
| *OsDRM2-1* | [AK065247](http://www.tigr.org/tigr-scripts/osa1_web/gbrowse/rice/?name=LOC_Os01g42630) | atcagcgttccagtttgt | tcgttgtgctttgggtag |
| *OsDRM2-2* | [AK063482](http://www.tigr.org/tigr-scripts/osa1_web/gbrowse/rice/?name=LOC_Os05g50290) | cagattcatgcgccctca | ggctacccacggaaacac |
| *OsDME* | AK241415 | agttcatagacttggtgct | ctaaaccgattggtgaga |
| *OsDDM1a* | AB177378 | ttaggcttggctctgtct | cggatgaaggaggatgta |
| *OsDDM1b* | AB177379 | cagccgaagtaagattga | cggatgaaggaggatgta |
